# LRRK2 Kinase Inhibitor Rejuvenates Oxidative Stress-Induced Cellular Senescence in Neuronal Cells

**DOI:** 10.1155/2021/9969842

**Published:** 2021-07-08

**Authors:** Dong Hwan Ho, Daleum Nam, Mi Kyoung Seo, Sung Woo Park, Wongi Seol, Ilhong Son

**Affiliations:** ^1^InAm Neuroscience Research Center, Sanbon Medical Center, Wonkwang University, Sanbon-ro, Gunpo-si, Gyeonggido, Republic of Korea; ^2^Paik Institute for Clinical Research, College of Medicine, Inje University, Republic of Korea; ^3^Department of Health Science and Technology, Graduate School of Inje University, Busanjin-gu, Busan, Republic of Korea; ^4^Department of Neurology, Sanbon Medical Center, Wonkwang University, Sanbon-ro, Gunpo-si, Gyeonggido, Republic of Korea

## Abstract

**Background:**

Leucine-rich repeat kinase 2 (LRRK2) plays a critical role in the pathogenesis of Parkinson's disease (PD). Aging is the most critical risk factor for the progression of PD. The correlation between aging and cellular senescence has been established. Cellular senescence is correlated with the dysregulation of the proteolytic pathway and mitochondrial dysfunction, which are also associated with the aggregation of *α*-synuclein (*α*-syn).

**Methods:**

Human dopaminergic neuron-like cells (differentiated SH-SY5Y cells) were treated with rotenone in the presence or absence of the LRRK2 kinase inhibitor GSK2578215A (GSK-KI) for 48 h. The markers of cellular senescence, including p53, p21^Waf1/Cip1^ (p21), *β*-galactosidase (*β*-gal), Rb phosphorylation, senescence-associated (SA) *β*-gal activity, and lysosomal activity, were examined. The dSH cells and rat primary cortical neurons were treated with *α*-syn fibrils 30 min before treatment with rotenone in the presence or absence of GSK-KI for 48 h. Mice were intraperitoneally injected with rotenone and MLi-2 (LRRK2 kinase inhibitor) once every two days for two weeks.

**Results:**

Rotenone upregulated LRRK2 phosphorylation and *β*-gal levels through the activation of the p53-p21 signaling axis and downregulated Rb phosphorylation. Additionally, rotenone upregulated SA *β*-gal activity, reactive oxygen species levels, and LRRK2 phosphorylation and inhibited lysosome activity. Rotenone-induced LRRK2 upregulation impaired the clearance of *α*-syn fibrils. Treatment with LRRK2 inhibitor mitigated rotenone-induced cellular senescence and *α*-syn accumulation.

**Conclusions:**

Rotenone-induced upregulation of LRRK2 kinase activity promoted cellular senescence, which enhanced *α*-syn accumulation. However, the administration of an LRRK2 kinase inhibitor rejuvenated rotenone-induced cellular senescence.

## 1. Introduction

Parkinson's disease (PD), which is the most common neurodegenerative disease, is characterized by impaired motor control [1]. Several genetic and environmental factors contribute to the pathogenesis of PD [2–5]. LRRK2, a major genetic risk factor for PD, exhibits both GTPase and kinase activities [6]. The LRRK2 G2019S mutant, which exhibits enhanced kinase activity, promotes PD progression [7]. The risk of developing PD in patients harboring the G2019S mutant increases with age [8] as aging is associated with the progression of neurodegenerative diseases [9]. Previously, we had reported that LRRK2 kinase activity promoted cellular senescence and inhibited the degradation of alpha-synuclein (*α*-syn) aggregates [10]. *α*-Syn is a major component of Lewy bodies (LB) or Lewy neurites, which are the postmortem markers of PD. Impaired degradation of *α*-syn results in its aggregation [11].

Cellular senescence impairs the protein degradation machinery [12–14]. Exposure to low-dose rotenone, which is reported to induce PD, promotes cellular senescence in the human trabecular meshwork cell line [15]. Additionally, rotenone upregulates LRRK2 kinase activity in the neurons [16]. Therefore, we hypothesized that rotenone promotes cellular senescence through the activation of LRRK2 kinase and consequently enhances *α*-syn aggregation.

## 2. Methods and Materials

### 2.1. Cell Culture and Treatment

The human neuroblastoma cell line (SH-SY5Y cells) was cultured in growth medium (Dulbecco's modified Eagle's medium (DMEM; 10-013-CV; Corning cellgro, Thermo Fisher Scientific, Waltham, MA, USA) supplemented with 10% fetal bovine serum (FBS, 35-010-CV, Corning cellgro), 1% penicillin/streptomycin (P/S, 10378-016, Gibco, Thermo Fisher Scientific), and 1% mycoplasma removal reagent (KOMA)) at 37°C in a 5% CO_2_ incubator (Thermo Fisher Scientific). The differentiated SH-SY5Y cells (dSH cells) were obtained after treatment with 10 *μ*M all-*trans* retinoic acid (R2625-100MG, Sigma-Aldrich, St. Louis, MO, USA) prepared in growth medium once every two days for seven days. On day 7 postretinoic acid treatment, the cells were cotreated with rotenone (1 *μ*M) and GSK2572815A (GSK-KI; LRRK2 kinase inhibitor; 1 *μ*M) prepared in growth medium for 48 h. Dimethyl sulfoxide (DMSO) was used as a vehicle for rotenone and GSK-KI. The *α*-syn fibrils were purified as described previously [10]. The rotenone- and GSK-KI-treated cells were treated with *α*-syn fibrils (70 nM). The rat primary cortical neurons were treated with rotenone (1 *μ*M), GSK-KI (1 *μ*M), and *α*-syn fibril (70 nM) for 48 h. The treatment conditions for the rat primary cortical neurons were identical to those employed for dSH. The cells were washed twice with ice-cold phosphate-buffered saline (PBS) and lysed in 1x sample buffer (50 mM Tris-HCl (pH 6.8), 2% sodium dodecyl sulfate, 10% glycerol, 1% *β*-mercaptoethanol, and 0.02% bromophenol blue).

### 2.2. Isolation and Culture of Primary Cortical Neurons from E16 Rat Embryos

Pregnant rats were euthanized with CO_2_ gas. The uterus was dissected, and the fetuses from the embryonic sacs were placed in ice-cold HBSS-/- (14175-079, Gibco, Thermo Fisher Scientific). The skull was peeled using forceps, and the cortices were dissected from the whole brain. After the removal of the meninges, the cortices were incubated with 0.25% Trypsin-EDTA (25200-056, Gibco, Thermo Fisher Scientific) at 37°C for 20 min in a water bath. Next, the cortices were incubated with 40 *μ*g/mL DNase I (DN25, Sigma-Aldrich), vortexed gently, and incubated for 5 min in a 37°C water bath. Most of the supernatant was removed by suction, and the samples were incubated with a serum inhibitor containing MEM (11090-08, Gibco, Thermo Fisher Scientific), 1.5% DMEM, 5% FBS, 2.5 mg/mL bovine serum albumin (BSA; A7906, Sigma-Aldrich), and 2.5 mg/mL trypsin inhibitor (T9253, Sigma-Aldrich). The serum inhibitor was removed, and the cell pellet was resuspended in 10 times its volume with the growth medium. The composition of the growth medium was as follows: neurobasal medium (21103-049, Corning cellgro, Thermo Fisher Scientific), 2% B-27 (17504044, Corning cellgro, Thermo Fisher Scientific), and 1x GlutaMAX-1 (35050-061, Corning cellgro, Thermo Fisher Scientific). The isolated rat primary cortical neuron cells (3 × 10^5^/well) were seeded in a 12-well plate (SPL30012, SPL Life Sciences, Pochoen, South Korea) and cultured in the growth medium at 37°C in a 5% CO_2_ incubator for 24 h. The culture medium was replaced with growth medium supplemented with 0.2 mg/mL of 5-fluoro-2′-deoxyuridine (F0503, Sigma-Aldrich) and 96 *μ*g/mL of uridine (U3750, Sigma-Aldrich) to inhibit mitosis. On day 6, the cells were treated with 1 *μ*M rotenone, 1 *μ*M GSK-KI, and 70 nM *α*-syn fibrils for 48 h and harvested with 1x sample buffer.

### 2.3. Western Blot

Western blotting was performed as described previously [17]. The following antibodies were used for the western blotting: rabbit anti-LRRK2 phospho-S935 monoclonal (ab133450, Abcam, Cambridge, UK), rabbit anti-LRRK2 phospho-S1292 monoclonal (ab203181, Abcam), mouse anti-LRRK2 monoclonal (N241A/34, NeuroMab, UC Davis, CA, USA), mouse anti-p53 monoclonal (for human p53: sc-126; for mouse p53: sc-393031; Santa Cruz Biotechnology, Dallas, TX, USA), mouse anti-p21^Waf1/Cip1^ (p21) monoclonal (CM5131, ECM Biosciences, Versailles, KY, USA), mouse anti-Rb monoclonal (#9309S, Cell Signaling Technology (CST), Danvers, MA, USA), anti-phosphoRb (Ser807/811) (#9308S, CST), mouse anti-*β*-galactosidase monoclonal (sc-377257, Santa Cruz Biotechnology), mouse anti-p62 monoclonal (ab56416, Abcam), mouse anti-*β*-actin monoclonal (sc-47778; Santa Cruz Biotechnology), rabbit anti-LC3B polyclonal (#2775S; CST), mouse anti-*α*-syn (clone 42) monoclonal (610786; BD Biosciences, San Jose, CA, USA), anti-phospho-threonine-arginine (#2351S, CST), anti-p53 (SC-99, Santa Cruz Biotechnology), anti-LaminB (SC-6216, Santa Cruz Biotechnology), goat peroxidase-conjugated AffiniPure anti-mouse IgG (H+L) (#115-035-003; Jackson Immunoresearch Laboratories Inc., West Grove, PA, USA), and goat peroxidase-conjugated AffiniPure anti-rabbit IgG (H+L) (#111-035-144; Jackson Immunoresearch Laboratories Inc.) antibodies. Immunoreactive signals in the nitrocellulose membrane were developed with Luminata Crescendo Western HRP (#WBLUR0500, Merck & Co., Inc., Kenilworth, NJ, USA), and the images were captured with a MicroChemi 4.2 camera (Shimadzu, Kyoto, Japan).

### 2.4. Proximity Ligation Assay (PLA) and Immunofluorescence (IF)

PLA was performed using Duolink® In Situ Detection Reagents Green (DUO92014-100RXN, Sigma-Aldrich), Duolink® In Situ PLA® Probe Anti-Mouse MINUS antibody (DUO92002-100RXN, Sigma-Aldrich), and Anti-Rabbit Plus antibody (DUO92004-100RXN, Sigma-Aldrich), following the manufacturer's instructions. The SH-SY5Y cells seeded in 96-well black plates (655077, Greiner Bio-One, Kremsmünster, Austria) were differentiated, and the differentiated cells were treated with rotenone and GSK-KI on day 7. The cells were rinsed twice with ice-cold Dulbecco's PBS (DPBS), fixed with 4% paraformaldehyde for 30 min at room temperature (RT), and rinsed thrice with ice-cold DPBS. Next, the cells were permeabilized using 0.1% Triton X-100 prepared in DPBS. After washing thrice with cold DPBS, the cells were incubated with a drop of blocking solution at 37°C for 1 h. The blocking solution was removed, and the cells were incubated with antibodies (50 *μ*L) at 37°C for 3 h. The antibody mixture was comprised of an antibody diluent from the kit (antibodies used for PLA: mouse anti-LRRK2 monoclonal (1 : 20) antibody and rabbit anti-LRRK2 phospho-S1292 monoclonal (1 : 20) antibody; antibody used for the IF assay: chicken anti-*β*-gal polyclonal antibody (1 : 400, ab9361, Abcam)). In the IF assay, the antibody was added throughout the entire process until the PLA application step. The samples were then washed twice with 1x wash buffer A for 5 min. To prepare the probe solution, the Duolink® In Situ PLA® Probe Anti-Mouse Minus and Anti-Rabbit Plus were mixed with the antibody diluent (1 : 5). For the IF assay, the anti-*β*-gal antibody was also added (1 : 400) to the mixture. The cells were incubated with 50 *μ*L of the probe solution at 37°C for 1 h. Next, the cells were washed twice with 1x wash buffer A for 5 min. For the IF assay, ligase (40 : 1) and anti-*β*-gal antibody (400 : 1) were mixed in the ligation buffer. The cells were incubated with the ligation solution (50 *μ*L) at 37°C for 30 min and washed twice with 1x wash buffer A for 5 min. For the IF assay, polymerase (1 : 40) was incubated with Goat Anti-Chicken IgY H+L (Alexa Fluor® 594; ab150172) secondary antibody (1 : 500) for 100 min at 37°C. The cells were washed twice with 1x wash buffer B for 10 min, followed by washing with 0.01x wash buffer B for 1 min. To stain the nucleus, the cells were incubated with Hoechst 33342 (1 : 1000; 62249, Thermo Fisher Scientific) prepared in DPBS at RT for 10 min. The images of the cells in the 96-well black plates were captured using a confocal laser scanning microscope (LSM 700, Carl Zeiss, Jena, Germany).

### 2.5. Immunoprecipitation

The whole-cell lysates of dSH cells treated with rotenone (1 *μ*M) and GSK-KI (1 *μ*M) were prepared using lysis solution (PBS, 1% Triton X-100, 1x protease inhibitor cocktail, and 1x phosphatase inhibitor cocktail). The lysates were centrifuged at 4,000*g* and 4°C for 5 min, and the supernatant was incubated with the anti-p53 antibody (554293, BD Biosciences) and Pierce™ Protein G Agarose (20398, Thermo Fisher Scientific) resuspended in the lysis solution for 12 h. The beads were washed twice with the lysis solution, and the bead-protein complex was denatured with 1x sample buffer.

### 2.6. Nuclear Fractionation

The nuclear fraction was isolated using NE-PER™ Nuclear and Cytoplasmic Extraction Reagents (78833, Thermo Fisher Scientific), following the manufacturer's instructions.

### 2.7. mRNA Preparation and Quantitative Real-Time Polymerase Chain Reaction (qRT-PCR)

mRNA was isolated from rotenone/GSK-KI-treated dSH cells using the RNeasy Plus Mini kit (74134, QIAGEN, Hilden, Germany). Next, mRNA was reverse transcribed into complementary DNA (cDNA) using the TOPscript™ cDNA Synthesis Kit (EZ005S, Enzynomics, Daejeon, Republic of Korea). cDNA was subjected to qRT-PCR using TOPreal™ qPCR 2x PreMIX (SYBR Green with low ROX, UDG plus) (RT500M, Enzynomics). The following primers were used for qRT-PCR: human p21, 5′-ATG AAA TTC ACC CCC TTT CC-3′ (forward) and 5′-CCC TAG GCT GTG CTC ACT TC-3′ (reverse); human p16^INK4^ (p16), 5′-CCC AAC GCA CCG AAT AGT TAC-3′ (forward) and 5′-CAC GGG TCG GGT GAG AGT-3′ (reverse). qRT-PCR was performed on the Mic qPCR cycler (MIC-4, Bio Molecular Systems, Upper Coomera, Australia).

### 2.8. Measurement of Senescence-Associated (SA) *β*-Galactosidase (*β*-Gal) Activity, Reactive Oxygen Species (ROS), and Lysosome Activity

At 30 min posttreatment, live dSH cells were stained with 2 *μ*M GlycoGREEN™-*β*Gal (GC611, Goryo Chemical Inc., Hokkaido, Japan), 5 *μ*M 2′,7′-dichlorofluorescein diacetate (DCFDA; 287810, Calbiochem, San Diego, CA, USA), 2 *μ*M LysoSensor™ Blue DND-167 (L7533, Invitrogen, Carlsbad, CA, USA), and 1 *μ*M Hoechst 33342 for examining SA *β*-gal activity, ROS, active lysosomes, and nucleus, respectively. The stained cells were mounted with ProLong™ Diamond Antifade Mountant (P36965, Invitrogen) and imaged using a confocal laser scanning microscope. Differential interference contrast images were obtained using a confocal laser scanning microscope.

### 2.9. SA *β*-Gal Activity Assay

The SA *β*-gal activity was examined using a 96-well cellular senescence assay kit (CBA-213, Cell Biolabs Inc., San Diego, CA, USA), following the manufacturer's instructions.

### 2.10. Mouse Handling and Drug Administration

Mice were handled as described previously [18] according to the guidelines of the Dankook Animal Ethics Committee (Dankook IACUC, 18-026). C57BL/6J male mice aged 16 weeks were intraperitoneally injected with rotenone (0.75 mg/kg bodyweight) and MLi-2 (an LRRK2 kinase inhibitor; 1 mg/kg bodyweight) once every two days for two weeks.

### 2.11. Rotarod Test and Brain Tissue Preparation

On day 14 posttreatment, the mice were placed on a rotatable cylinder-shaped rod. The time required to fall to the floor was recorded. The details of the rotarod test are described elsewhere [18]. Next, the mice were euthanized and transcardially perfused with ice-cold HBSS containing Ca^2+^ and Mg^2+^. The midbrain was dissected with microscissors and tweezers. The tissues were lysed in PBS containing 1% Triton X-100, 1x Xpert Protease Inhibitor Cocktail Solution (P3100, GenDEPOT, Katy, TX, USA), and 1x Xpert Phosphatase Inhibitor Cocktail Solution (P3200, GenDEPOT). The samples were homogenized using a Pellet Pestle Cordless Motor (Sigma-Aldrich). The supernatant was subjected to western blotting, SA *β*-gal activity assay, and enzyme-linked immunosorbent assay (ELISA).

### 2.12. ELISA for Oligomeric *α*-Syn

Previously, we had established a sandwich ELISA for the analysis of fibrillar *α*-syn oligomers using an antibody that recognizes the filamentous conformation of *α*-syn aggregates [17]. The crude midbrain lysates were subjected to ELISA, and the *α*-syn aggregates were quantified using *α*-syn fibril standards.

### 2.13. Image and Data Analyses

The intensities of PLA and live-cell staining were measured using Zen 2012 (Carl Zeiss). Densitometry of the target proteins was performed using Multi Gauge (Fujifilm, Tokyo, Japan). All datasets were analyzed and graphed using Prism 8 (GraphPad Software, San Diego, CA, USA). Data from experiments performed using dSH cells and rat primary cortical neurons were analyzed using two-way analysis of variance (ANOVA), followed by Bonferroni's post hoc test (*n* = 3). Meanwhile, the data obtained from mouse experiments, live-cell staining, and PLA in the dSH cells were analyzed using two-way ANOVA, followed by Tukey's post hoc test (*n* > 3). All data are represented as mean ± standard error of mean. ^∗^*p* < 0.05, ^∗∗^*p* < 0.01, ^∗∗∗^*p* < 0.001, ^∗∗∗∗^*p* < 0.0001, and n.s. = not significant.

## 3. Results

### 3.1. Rotenone Upregulates LRRK2 Kinase Activity and Cellular Senescence in dSH Cells

**Figure 1:**
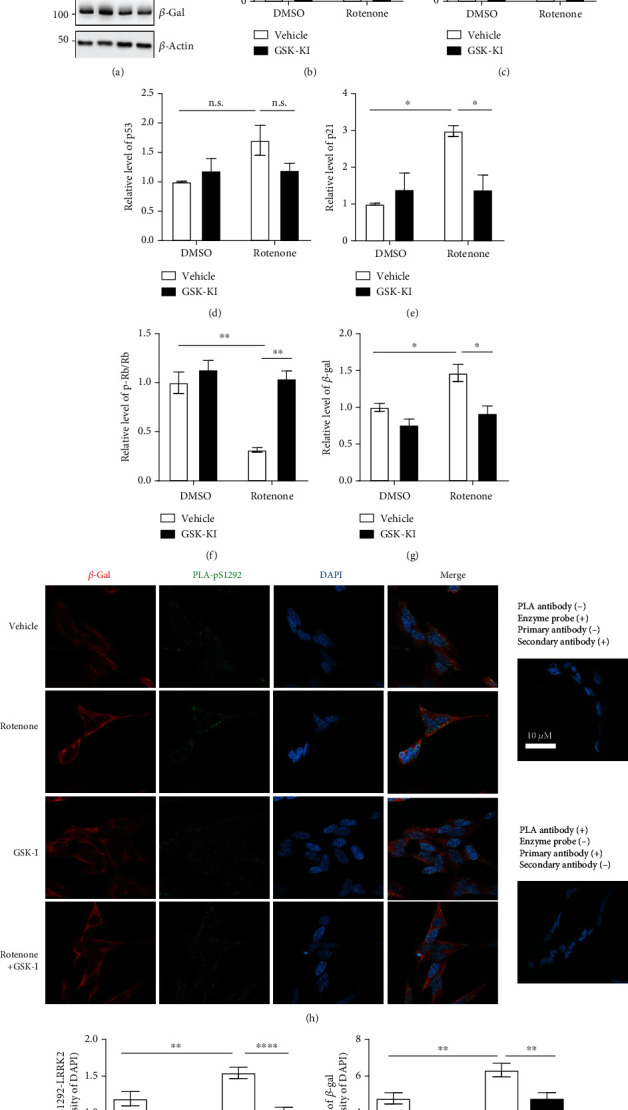
Rotenone-induced LRRK2 kinase activation promotes cellular senescence in the differentiated human neuroblastoma cell line. Western blotting analysis of dSH cells treated with 1 *μ*M rotenone or 1 *μ*M GSK2578215A (GSK-KI), an LRRK2 kinase inhibitor, for 48 h using antibodies against the target proteins (a). The densities of the proteins were normalized to those of *β*-actin (b–g). *n* = 3. (h) dSH cells treated with 1 *μ*M rotenone or 1 *μ*M GSK-KI for 48 h were subjected to proximity ligation assay (PLA) of phospho-S1292 LRRK2 and total LRRK2 (PLA-pS1292) and immunofluorescence (IF) analysis of *β*-galactosidase. Nuclei were stained with Hoechst 33342. The controls used in PLA and IF staining revealed the validity of the results (right two panels). The intensities of PLA-pS1292 (i) and *β*-galactosidase (j) were normalized to those of 4′,6-diamidino-2-phenylindole. *n* = 4; number of cells = 12–17.

**Figure 2:**
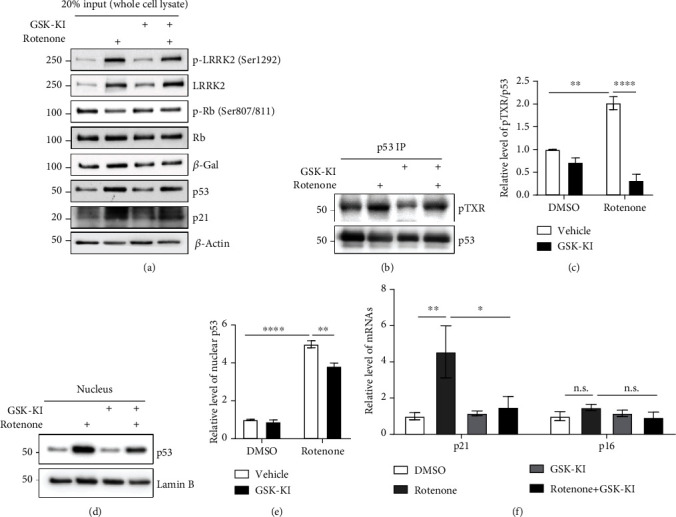
LRRK kinase inhibitor inhibits the activation of p53 in the differentiated human neuroblastoma cell line. (a) Whole-cell lysates (20%), which were used as the input of immunoprecipitation (IP), and nuclear fraction were collected. The analysis revealed that 20% input exhibited results similar to those observed in Figure 1. (b, c) The IP samples were denatured and subjected to western blotting. The TXR site phosphorylation levels in p53 were normalized to total p53 levels. *n* = 3. (d–e) Total p53 levels in the nucleus were examined using western blotting. LaminB was used for the normalization of nuclear p53 levels. *n* = 3. (f) The mRNA levels of p21 and p16 in the treatment group were normalized to those in the vehicle- (dimethyl sulfoxide-) treated group. *n* = 3.

Previous studies have reported that rotenone upregulates LRRK2 kinase activity by upregulating LRRK2 expression [16, 19]. However, the rates of apoptosis were higher than those of other cellular senescence-associated phenotypes in these studies due to the use of high rotenone concentration. Hence, the cells were treated with a low rotenone concentration (1 *μ*M) for a prolonged duration (48 h) as reported previously [15]. Rotenone upregulated the LRRK2 levels. However, cotreatment with rotenone and GSK-KI did not result in the upregulation of LRRK2 levels (Figures 1(a) and 1(b)). Treatment with GSK-KI mitigated the rotenone-induced S1292 phosphorylation of LRRK2 (Figures 1(a)–1(c)). Similarly, treatment with GSK-KI nonsignificantly mitigated the rotenone-induced upregulation of p53 levels (Figures 1(a) and 1(d)). LRRK2 has been reported to phosphorylate p53 at the TXR site [20]. Treatment with rotenone enhanced the nuclear localization of p53, which was mitigated upon cotreatment with GSK-KI (Figures 2(a)–2(e)). Additionally, GSK-KI mitigated the rotenone-induced upregulation of p21 (Figures 1(a) and 1(e)). Rotenone downregulated the phosphorylation of Rb, which is involved in the last stage of the senescent p53-p21 pathway. Treatment with GSK-KI mitigated the rotenone-induced phosphorylation of Rb (Figures 1(a) and 1(f)). As the p53-p21 pathway is not the only signaling pathway mediating senescence, the mRNA levels of p21 and p16 were examined. Treatment with GSK-KI mitigated the rotenone-induced upregulation of p21. The p16 mRNA levels were not significantly different between rotenone-treated and rotenone/GSK-KI-treated groups (Figure 2(f)). Rotenone upregulated the activity of *β*-gal, a senescence marker, which was mitigated upon cotreatment with GSK-KI (Figures 1(a) and 1(g)). Moreover, the results of PLA of pS1292 LRRK2 and total LRRK2 in the dSH cells revealed that rotenone increased the *β*-gal levels and the LRRK2 kinase activity (Figures 1(h)–1(j)). These results indicate that rotenone promotes cellular senescence through the p53, p21, Rb, and *β*-gal pathways by upregulating the LRRK2 levels and its kinase activity and that LRRK2 inhibition mitigated rotenone-induced cellular senescence in the dSH cells.

### 3.2. Cellular Senescence Impairs *α*-Syn Degradation in dSH Cells and Rat Primary Cortical Neurons by Upregulating the Expression of LRRK2 Kinase

**Figure 3:**
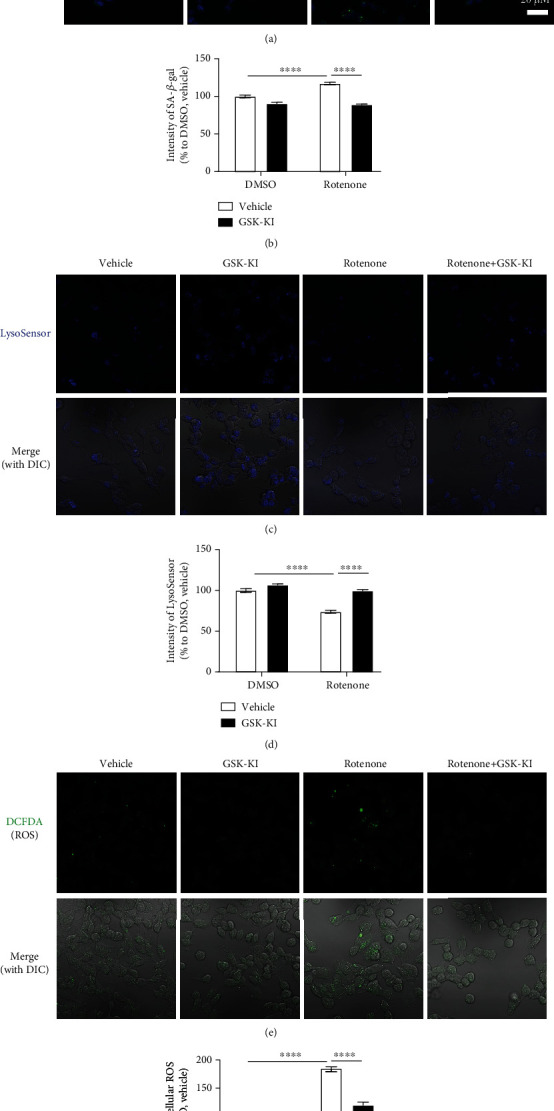
Senescence promotes lysosomal dysfunction by upregulating LRRK2 kinase activity in the differentiated human neuroblastoma cell line. (a, b) GlycoGREEN (Green) staining of the live dSH cells. The staining intensity of GlycoGREEN was normalized to that of Hoechst 33342. The normalized intensity in the treatment group was expressed in terms of percentage (%) relative to that in the vehicle- (dimethyl sulfoxide-) treated group. *n* = 4; number of cells = 15–40. (c, d) The active lysosomes, which are associated with acidic conditions, were stained with LysoSensor Blue DND-167. The staining intensity was expressed in terms of percentage (%) relative to that in the vehicle-treated group. *n* = 4; number of cells = 17–38. (e, f) The levels of reactive oxygen species in the dSH cells treated with rotenone (1 *μ*M) and GSK-KI (1 *μ*M) were measured using 2′,7′-dichlorofluorescein diacetate (DCFDA). The DCFDA intensity in the treatment groups was normalized to that in the vehicle-treated groups. The estimated intensities of DCFDA were expressed in terms of percentage (%). *n* = 4; number of cells = 25–44.

**Figure 4:**
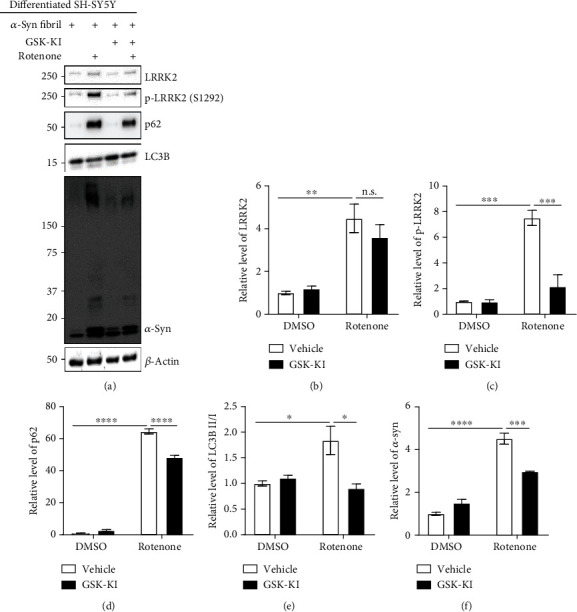
LRRK2 kinase inhibitor mitigates rotenone-induced impaired autophagy and enhanced *α*-synuclein accumulation in the differentiated neuroblastoma cell line. dSH cells treated with rotenone, GSK-KI, and *α*-synuclein fibril (70 nM) were subjected to western blotting (a). The densities of target proteins were normalized to those of *β*-actin (b–d and f). The density of LC3B II was normalized to that of LC3B I (e). *n* = 3.

**Figure 5:**
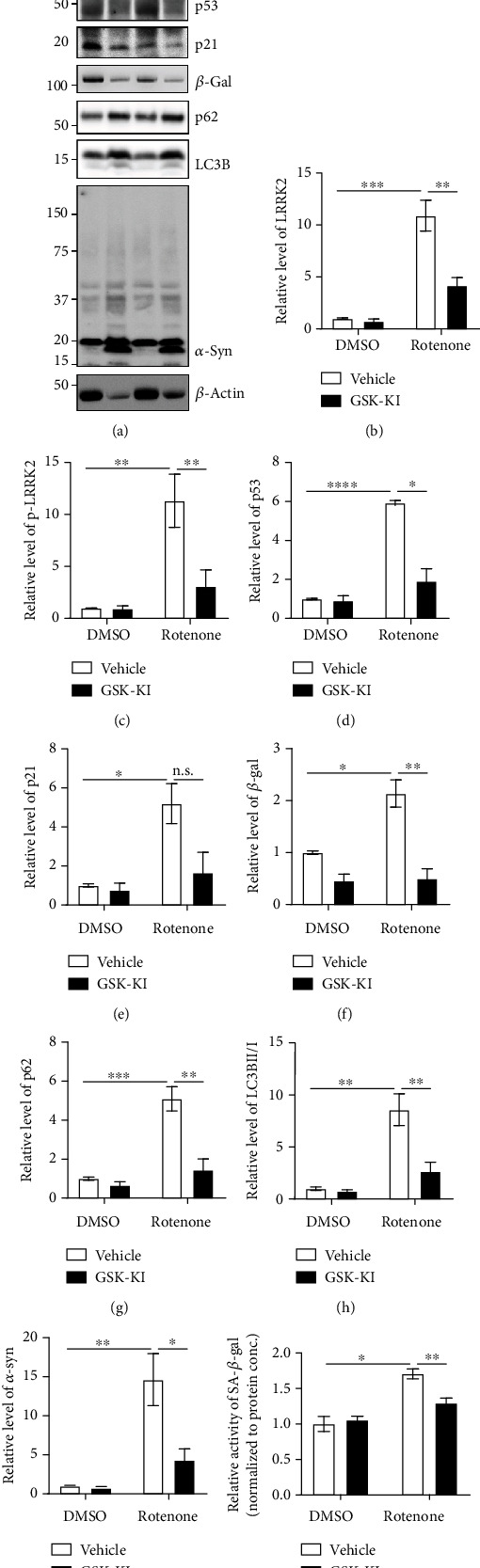
LRRK2 kinase inhibition mitigates rotenone-induced cellular senescence and promotes the autophagic clearance of *α*-synuclein in the rat primary cortical neurons. The experiments performed using dSH treated with *α*-synuclein fibril (70 nM), rotenone (1 *μ*M), and GSK-KI (1 *μ*M) for 48 h were repeated with rat primary cortical neurons. Immunoreactive signals in the western blot (a) were visualized using antibodies against the target proteins. The densities of all proteins, except LC3B II/I, were normalized to those of *β*-actin (b–i). (j) The lysates of (a) were subjected to the senescence-associated *β*-galactosidase activity assay. *n* = 3.

Cellular senescence impairs the activity of the autophagy-lysosomal pathway [14, 21]. Hence, the lysosomal activity (LysoSensor) was examined along with senescence markers, SA *β*-gal activity, and ROS levels under identical experimental conditions (Figure 1). Rotenone increased the SA *β*-gal activity (Figures 3(a) and 3(b)) and decreased the lysosomal activity (Figures 3(c) and 3(d)), which were mitigated upon cotreatment with GSK-KI. Additionally, GSK-KI mitigated rotenone-induced upregulation of ROS levels, which indicated that rotenone promotes senescence by inducing oxidative stress (Figures 3(e) and 3(f)). Previously, we had demonstrated that LRRK2-mediated p53 phosphorylation, as well as the LRRK2 G2019S mutant, promoted the accumulation of *α*-syn [10]. The effect of cellular senescence on the aggregation of *α*-syn, which exacerbates the progression of PD, was examined using *α*-syn fibrils. Rotenone upregulated the levels of p62 and LC3B II/I, which represent the suspension of the autophagy flux, *α*-syn aggregates (Figures 4(a), 4(d), and 4(f)), and LRRK2 kinase activity (Figures 4(a)–4(c)) in the dSH cells. Treatment with GSK-KI mitigated the rotenone-induced upregulation of p62, LC3B II/I, and *α*-syn aggregates but did not affect the LRRK2 levels. Additionally, treatment with GSK-KI mitigated the rotenone-induced upregulation of SA *β*-gal and downregulation of lysosomal enzyme (cathepsin D) activities (Supplementary Figure 1). To determine the mechanism underlying rotenone-mediated LRRK2 kinase activation and cellular senescence, the experiments performed with dSH cells were repeated using rat primary cortical neurons. Rotenone and GSK-KI exerted similar effects on LRRK2 kinase activity (Figures 5(a)–5(c)), p53-p21 pathway activation (Figures 5(a), 5(d), and 5(e)), *β*-gal levels (Figures 5(a) and 5(f)), autophagy (Figures 5(a), 5(g), and 5(h)), *α*-syn aggregation (Figures 5(a) and 5(i)), and SA *β*-gal activity (Figure 5(j)) in the rat primary cortical neurons as those observed in the dSH cells. Thus, rotenone-induced cellular senescence promotes the upregulation of LRRK2 kinase and impairs autophagy-lysosomal activities, which leads to enhanced *α*-syn aggregation.

### 3.3. LRRK2 Kinase Inhibition Alleviates Rotenone-Induced Cellular Senescence in the Mouse Midbrain

**Figure 6:**
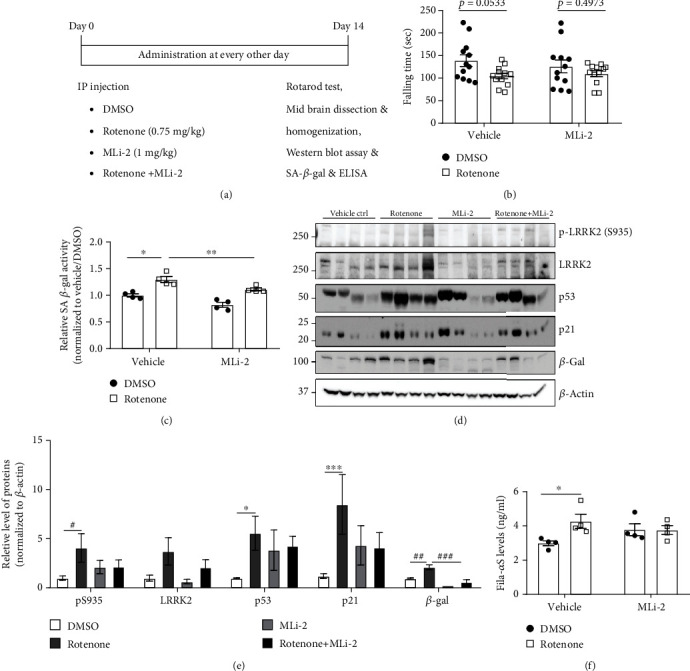
Rotenone promotes cellular senescence in the mouse midbrain by upregulating the expression of LRRK2 kinase. (a) A scheme of the mouse model experiment. (b) The measurements of falling time from the rotarod are represented. The falling times of each mouse in the experimental group (*n* = 4) were recorded thrice. The midbrain lysates were subjected to senescence-associated *β*-galactosidase activity assay (c), western blotting (d–e), and enzyme-linked immunosorbent assay for detecting filamentous oligomer *α*-synuclein (f). *n* = 4. ∗ indicates the results of two-way analysis of variance (ANOVA), followed by Tukey's post hoc test. # indicates the results of one-way ANOVA, followed by Tukey's post hoc test. ^##^*p* < 0.01 and ^###^*p* < 0.001.

To verify the *in vitro* findings, an *in vivo* assay was performed. Mice were intraperitoneally injected with rotenone (0.75 mg/kg bodyweight) and MLi-2 (1 mg/kg bodyweight), a blood-brain barrier-permeable LRRK2 kinase inhibitor, once every two days for two weeks (Figure 6(a)). In experiments performed using high rotenone concentrations, apoptosis was the predominant cellular senescence-associated phenotype. Hence, the dSH cells and rat primary neurons were treated with low concentrations of rotenone for a prolonged duration. Rotenone nonsignificantly decreased the locomotor activity of mice as evidenced by the decreased time of falling from the rotarod (Figure 6(b)). However, rotenone upregulated the SA *β*-gal activity in the midbrain, which was significantly mitigated upon cotreatment with MLi-2 (Figure 6(c)). Additionally, treatment with MLi-2 nonsignificantly mitigated the rotenone-induced activation of the LRRK2 kinase and p53-p21 pathway in the midbrain (Figures 6(d) and 6(e)). Furthermore, rotenone significantly upregulated the levels of *β*-gal, which was mitigated upon cotreatment with MLi-2. ELISA was performed to measure the levels of fibrillar *α*-syn oligomers in the midbrain lysate [17]. Treatment with MLi-2 mitigated the rotenone-induced upregulation of fibrillar *α*-syn oligomers (Figure 5(f)). Thus, the intraperitoneal administration of low-dose rotenone promoted cellular senescence in the mouse midbrain, which resulted in *α*-syn aggregation in the midbrain. Treatment with LRRK2 kinase inhibitor mitigated rotenone-induced cellular senescence in the midbrain.

## 4. Discussion

The mechanism underlying cellular senescence in the dopaminergic neurons has not been elucidated. Aging is a risk factor for PD. Hence, the correlation between aging and cellular senescence must be considered. Recent studies have suggested that PD-associated cellular senescence is related to the loss of cellular functions [14, 22, 23], such as the loss of LRRK2 function in the neurons resulting from deregulation of the autophagy-lysosomal pathway [24, 25]. Previously, we had demonstrated that the G2019S LRRK2 mutant impaired autophagy in a mouse dopaminergic cell line by phosphorylating leucine-tRNA synthetase [26]. An impaired autophagy-lysosomal pathway promotes *α*-syn aggregation [11, 27]. Hence, LRRK2 activity may mediate *α*-syn aggregation. The inhibition or prevention of *α*-syn aggregation using an anti-*α*-syn antibody is a potential therapeutic strategy for PD [28–30]. However, the anti-*α*-syn antibody did not exert a marked therapeutic effect on PD in humans. This may be due to the incomplete clearance of *α*-syn aggregates, including fibrils, protofibrils, and toxic oligomers. Conformation-specific antibodies for *α*-syn oligomers may not clear all toxic or LB-prone oligomers in the brain of patients with PD. LRRK2 kinase inhibition can activate the autophagy-lysosomal pathway and promote *α*-syn clearance. Hence, the coadministration of an LRRK2 kinase inhibitor and an anti-*α*-syn antibody exerts a synergistic therapeutic effect on PD.

The p53-p21 pathway is involved in several cellular processes, including apoptosis and senescence [31]. The phosphorylation of Rb is the gating step of the cellular senescence signaling pathway [32]. Additionally, the activation of p16 expression mediates cellular senescence [33]. The expression levels of p16 and p21 are upregulated in the dorsal root ganglion neurons of cisplatin-injected mice [34]. Additionally, postmortem analysis of patients with amyotrophic lateral sclerosis/motor neuron disease revealed that the expression of p16 and p21 was upregulated in the astrocytes of the frontal associated cortex. However, the neurons exhibited upregulation of p21 with undetectable levels of p16 [35]. The findings of this study indicated that cellular senescence was mainly mediated by the p53-p21 pathway rather than the upregulated p16 (Figures 1, 2, and 4). Inflammatory responses are also associated with senescence. Astrocytes are involved in neuroinflammation in the brain [36]. Astrocyte senescence contributes to the progression of cognitive abnormalities [37]. Thus, neuroinflammation may be related to senescence in astrocytes. Future studies must investigate the correlation between neuroinflammation and cellular senescence and its distinct signaling pathways.

**Figure 7:**
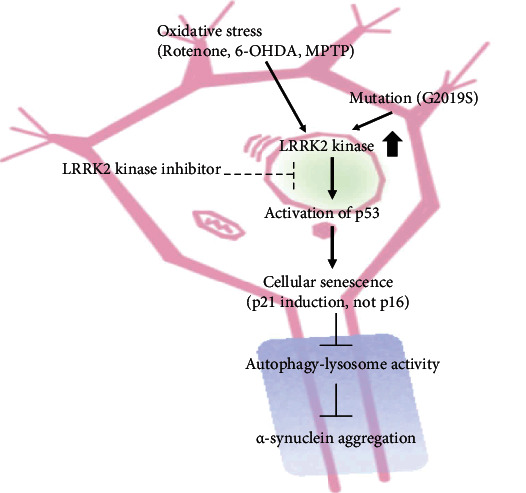
Role of LRRK2 kinase inhibitor in inducing cellular senescence during the progression of Parkinson's disease. A schematic representation of the findings of this study. The LRRK2 kinase inhibitor mitigates rotenone-induced cellular senescence, impaired autophagy-lysosomal pathway, and enhanced *α*-synuclein aggregation in the dopaminergic neurons.

In this study, rotenone upregulated LRRK2 kinase activity. However, this study did not demonstrate the effect of the LRRK2 kinase inhibitor on the G2019S mutant. Cellular senescence was examined after the transfection of the G2019S LRRK2 mutant in the dSH cells and rat primary neurons. The prolonged expression of G2019S LRRK2 in the dSH cells and rat primary neurons significantly increased *β*-gal levels and SA *β*-gal activity. Treatment of G2019S LRRK2-transfected cells with the LRRK2 kinase inhibitor or the expression of D1994A LRRK2 did not upregulate the *β*-gal levels and SA *β*-gal activity (Supplementary Figure 2). Previously, we had reported that the levels of *β*-gal and *α*-syn aggregates in the brain lysate of 24-week-old G2019S-expressing mice were higher than those in the age-matched littermates [10]. Treatment with an LRRK2 inhibitor did not markedly mitigate low-dose rotenone-induced cellular senescence in mice. However, pharmacological advances may enhance the therapeutic effect of LRRK2 kinase inhibitors in mice (Figure 6). Future studies must focus on improving the pharmacological effects and safety profile of LRRK2 kinase inhibitors. The administration of an LRRK2 kinase inhibitor can serve as a feasible therapeutic strategy for PD with high LRRK2 kinase activity as it can mitigate oxidative stress or the toxic effects of LRRK2 mutants (Figure 7). LRRK2 kinase activity in the human biofluids, such as the serum, cerebrospinal fluid, and urine can serve as a prognostic biomarker to determine the optimal treatment for patients with PD exhibiting enhanced LRRK2 kinase activity.

## 5. Conclusions

The findings of this study suggest that treatment with LRRK2 kinase inhibitors mitigates cellular senescence induced by mild and long-term dosing of rotenone. This study demonstrated the critical role of LRRK2 in the p53-p21 pathway, which mediates cellular senescence, and provided novel insights for developing PD therapy using LRRK2 kinase inhibitors.

## Data Availability

The datasets generated and/or analyzed during the present study are available from the corresponding authors upon reasonable request.

## Supplementary Materials

Supplementary Materials Supplementary Figure 1. Rotenone-mediated LRRK2 kinase activation and cellular senescence in the presence of *α*-synuclein fibrils in the differentiated SH-SY5Y cells (dSH cells; human neuroblastoma cell line). (A–B) dSH cells treated with rotenone (1 *μ*M), GSK-KI (1 *μ*M), and *α*-synuclein fibril (70 nM) for 48 h were stained using the cellular senescence assay kit (CBA-230, CELL BIOLABS, INC.). The densities of the proteins were analyzed using Multi Gauge. The density was represented in terms of arbitrary unit (A.U.) (C) The cathepsin D activity was measured using the cathepsin D activity fluorometric assay kit (K143, BioVision, Milpitas, CA, USA). The cathepsin D activity in the treatment group was normalized to that in the vehicle control (dimethyl sulfoxide)-treated group. n = 4. Data are represented as mean ± standard error of mean. ∗∗∗∗*p* < 0.0001 (Two-way analysis of variance, followed by Tukey's post hoc test). Supplementary Figure 2. Inhibition of LRRK2 kinase mitigates the rotenone-induced activation of senescence-associated (SA) *β*-galactosidase in the differentiated SH-SY5Y cells and rat primary cortical neurons. (A) Ectopic expression of LRRK2 mutants in the dSH cells. The cells were transfected with vector (V), myc-tagged G2019S (GS), and myc-tagged D1994A (DA) using Lipofectamine^TM^ LTX reagent with PLUS^TM^ Reagent (15338100, Invitrogen) for 12 h on day 2 of differentiation of SH-SY5Y cells. The cells were then treated with GSK-KI (1 *μ*M) for six days. On day 8, the cells were lysed with lysis buffer and subjected to western blotting. (D) Ectopic expression of LRRK2 mutants in the rat primary cortical neurons. On day 2 of rat primary neuron cultures, the DNA plasmid was transfected into cells using Lipofectamin^TM^ LTX reagent with PLUS^TM^ Reagent for 16 h. The cells were then treated with GSK-KI (1 *μ*M) for five days. Ectopic LRRK2 exhibited a low transfection efficiency. The LRRK2 expression levels in the transfected cells were detected at day 5 for 48 h (48 h). (B, E) The levels of *β*-galactosidase were normalized to those of *β*-actin. The *β*-galactosidase levels in the vector-transfected group were used for the estimation. (C, F) Half of the lysates were used for the measurement of SA *β*-galactosidase activity. The fluorescence intensity was estimated based on the fluorescence intensity in the vector control-transfected group. Data are represented as mean ± standard error of mean. ∗*p* < 0.05, ∗∗*p* < 0.01, and ∗∗∗*p* < 0.001 (Two-way analysis of variance, followed by Bonferroni's post hoc test).
