# Supplementary material for: LRRK2 Kinase Inhibitor Rejuvenates Oxidative Stress-Induced Cellular Senescence in Neuronal Cells
Source: Oxid Med Cell Longev. 2021 Jul 8;2021:9969842. doi: 10.1155/2021/9969842 (PMC8282384; doi:10.1155/2021/9969842)
Supplement: Supplementary Materials — Supplementary Figure 1. Rotenone-mediated LRRK2 kinase activation and cellular senescence in the presence of α-synuclein fibrils in the differentiated SH-SY5Y cells (dSH cells; human neuroblastoma cell line). (A–B) dSH cells treated with rotenone (1 μM), GSK-KI (1 μM), and α-synuclein fibril (70 nM) for 48 h were stained using the cellular senescence assay kit (CBA-230, CELL BIOLABS, INC.). The densities of the proteins were analyzed using Multi Gauge. The density was represented in terms of arbitrary unit (A.U.) (C) The cathepsin D activity was measured using the cathepsin D activity fluorometric assay kit (K143, BioVision, Milpitas, CA, USA). The cathepsin D activity in the treatment group was normalized to that in the vehicle control (dimethyl sulfoxide)-treated group. n = 4. Data are represented as mean ± standard error of mean. ∗∗∗∗p < 0.0001 (Two-way analysis of variance, followed by Tukey's post hoc test). Supplementary Figure 2. Inhibition of LRRK2 kinase mitigates the rotenone-induced activation of senescence-associated (SA) β-galactosidase in the differentiated SH-SY5Y cells and rat primary cortical neurons. (A) Ectopic expression of LRRK2 mutants in the dSH cells. The cells were transfected with vector (V), myc-tagged G2019S (GS), and myc-tagged D1994A (DA) using LipofectamineTM LTX reagent with PLUSTM Reagent (15338100, Invitrogen) for 12 h on day 2 of differentiation of SH-SY5Y cells. The cells were then treated with GSK-KI (1 μM) for six days. On day 8, the cells were lysed with lysis buffer and subjected to western blotting. (D) Ectopic expression of LRRK2 mutants in the rat primary cortical neurons. On day 2 of rat primary neuron cultures, the DNA plasmid was transfected into cells using LipofectaminTM LTX reagent with PLUSTM Reagent for 16 h. The cells were then treated with GSK-KI (1 μM) for five days. Ectopic LRRK2 exhibited a low transfection efficiency. The LRRK2 expression levels in the transfected cells were detected at day 5 for 48 h (48 h). [file 9969842.f1.docx]

**Supplementary figures**

**LRRK2 kinase inhibitor rejuvenates oxidative stress-induced cellular senescence in neuronal cells**

Dong Hwan Ho^1, *^, Daleum Nam^1^, Mi Kyoung Seo^2^, Sung Woo Park^2, 3^, Wongi Seol^1^, Ilhong Son^1, 4, *^

*^1^InAm Neuroscience Research Center, Sanbon Medical Center, Wonkwang University, Sanbon-ro, Gunpo-si; ^2^Paik Institute for Clinical Research, College of Medicine, Inje University; ^3^Department of Health Science and Technology, Graduate School of Inje University, Busanjin-gu, Busan; ^4^Department of Neurology, Sanbon Medical Center, Wonkwang University, Sanbon-ro, Gunpo-si, Gyeonggido, Republic of Korea*

^*^Co-corresponding authors: Dong Hwan Ho (Tel: +82-31-390-2410, Fax: +82-31-390-2414, e-mail: ethan2887@gmail.com), Ilhong Son (Tel: +82-31-390-2486, Fax: +82-31-390-2414, e-mail: sonih@wku.ac.kr)

**
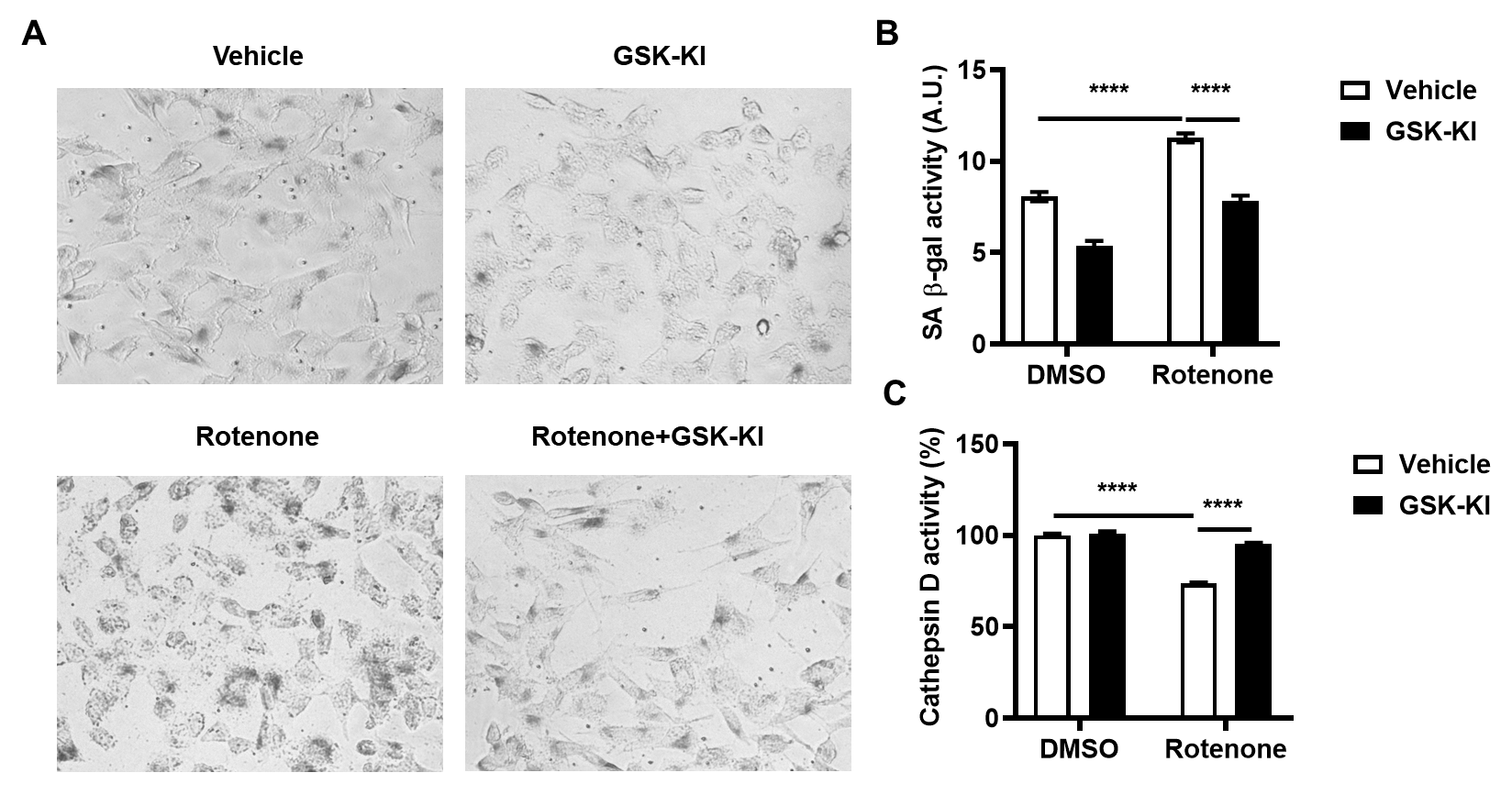
**

**Supplementary Figure 1.** Rotenone-mediated LRRK2 kinase activation and cellular senescence in the presence of α-synuclein fibrils in the differentiated SH-SY5Y cells (dSH cells; human neuroblastoma cell line). (A–B) dSH cells treated with rotenone (1 μM), GSK-KI (1 μM), and α-synuclein fibril (70 nM) for 48 h were stained using the cellular senescence assay kit (CBA-230, CELL BIOLABS, INC.). The densities of the proteins were analyzed using Multi Gauge. The density was represented in terms of arbitrary unit (A.U.) (C) The cathepsin D activity was measured using the cathepsin D activity fluorometric assay kit (K143, BioVision, Milpitas, CA, USA). The cathepsin D activity in the treatment group was normalized to that in the vehicle control (dimethyl sulfoxide)-treated group. n = 4. Data are represented as mean ± standard error of mean. *****p<*0.0001 (Two-way analysis of variance, followed by Tukey’s post hoc test).


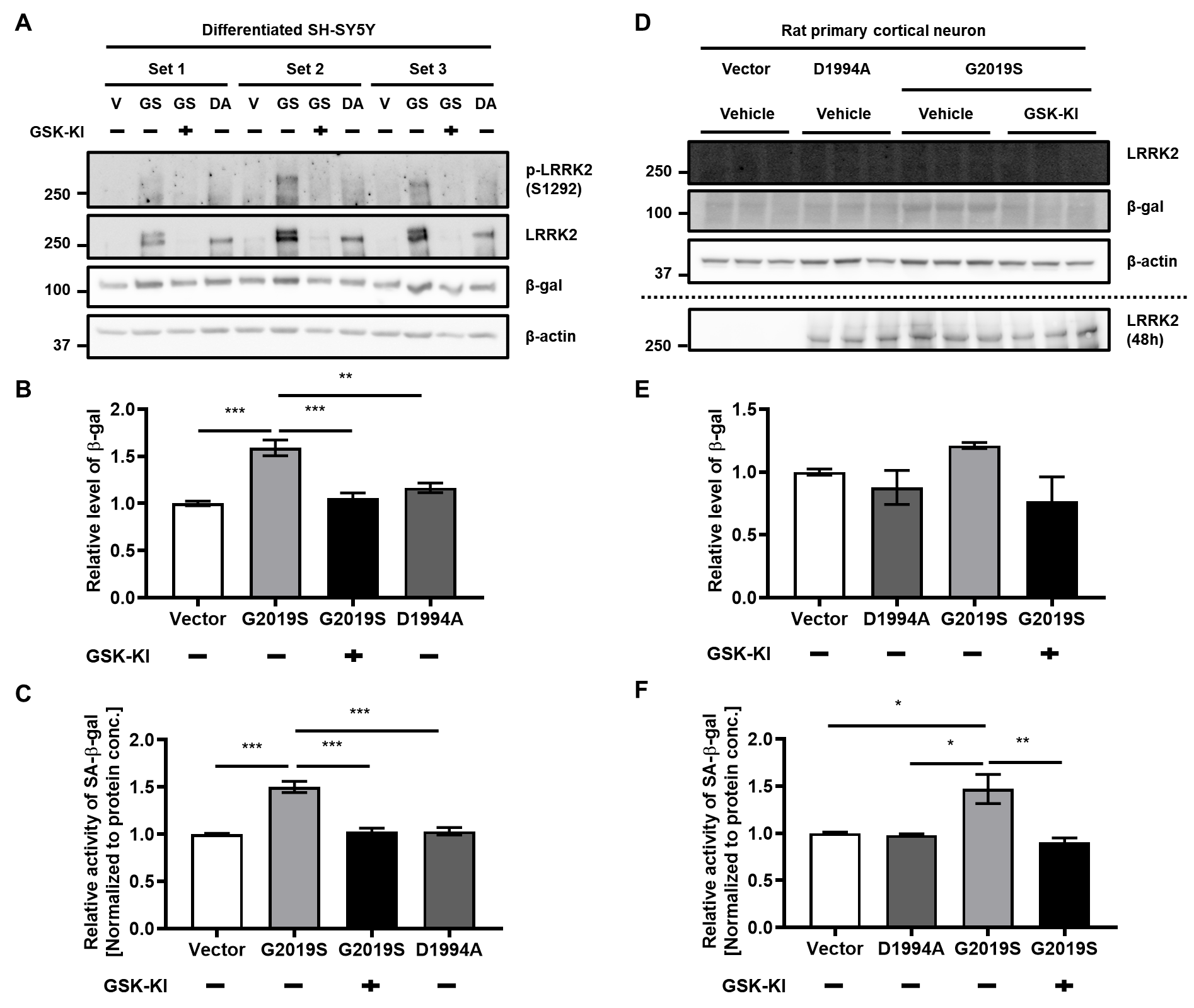


**Supplementary figure 2.** **Supplementary Figure 2.** Inhibition of LRRK2 kinase mitigates the rotenone-induced activation of senescence-associated (SA) β-galactosidase in the differentiated SH-SY5Y cells and rat primary cortical neurons. (A) Ectopic expression of LRRK2 mutants in the dSH cells. The cells were transfected with vector (V), myc-tagged G2019S (GS), and myc-tagged D1994A (DA) using Lipofectamine^TM^ LTX reagent with PLUS^TM^ Reagent (15338100, Invitrogen) for 12 h on day 2 of differentiation of SH-SY5Y cells. The cells were then treated with GSK-KI (1 μM) for six days. On day 8, the cells were lysed with lysis buffer and subjected to western blotting. (D) Ectopic expression of LRRK2 mutants in the rat primary cortical neurons. On day 2 of rat primary neuron cultures, the DNA plasmid was transfected into cells using Lipofectamin^TM^ LTX reagent with PLUS^TM^ Reagent for 16 h. The cells were then treated with GSK-KI (1 μM) for five days. Ectopic LRRK2 exhibited a low transfection efficiency. The LRRK2 expression levels in the transfected cells were detected at day 5 for 48 h (48 h). (B, E) The levels of β-galactosidase were normalized to those of β-actin. The β-galactosidase levels in the vector-transfected group were used for the estimation. (C, F) Half of the lysates were used for the measurement of SA β-galactosidase activity. The fluorescence intensity was estimated based on the fluorescence intensity in the vector control-transfected group. Data are represented as mean ± standard error of mean. **p<*0.05, ***p<*0.01, and ****p<*0.001 (Two-way analysis of variance, followed by Bonferroni’s post hoc test).
